# Integrating frailty interventions into existing care models: a comprehensive approach to enhancing patient outcomes in chronic disease management

**DOI:** 10.3389/fpubh.2024.1518774

**Published:** 2025-01-21

**Authors:** Izabella Uchmanowicz, Kenneth M. Faulkner, Paolo Iovino, Adrian Kwaśny, Stanisław Surma, Camilla Elena Magi, Grzegorz K. Jakubiak, Yari Longobucco, Dawid Janczak, Alina Rak-Pasikowska, Michał Czapla, Bartosz Uchmanowicz

**Affiliations:** ^1^Department of Nursing, Faculty of Nursing and Midwifery, Wroclaw Medical University, Wroclaw, Poland; ^2^Centre for Cardiovascular Health, Edinburgh Napier University, Edinburgh, United Kingdom; ^3^Stony Brook University School of Nursing, Stony Brook, NY, United States; ^4^Department of Health Sciences, University of Florence, Firenze, Italy; ^5^Institute of Dietetics, The Academy of Business and Health Science, Lodz, Poland; ^6^Department of Internal Medicine and Clinical Pharmacology, Medical University of Silesia, Medyków, Katowice, Poland; ^7^Department of Pharmacology, Faculty of Medical Sciences in Zabrze, Medical University of Silesia, Zabrze, Poland; ^8^Department of Minimally Invasive and Robotic Urology, University Center of Excellence in Urology, Wrocław Medical University, Wrocław, Poland; ^9^Division of Clinical Chemistry and Laboratory Haematology, Department of Medical Laboratory Diagnostics, Faculty of Pharmacy, Wroclaw Medical University, Wrocław, Poland; ^10^Department Division of Scientific Research and Innovation in Emergency Medical Service, Department of Emergency Medical Service, Faculty of Nursing and Midwifery, Wroclaw Medical University, Wroclaw, Poland; ^11^Group of Research in Care (GRUPAC), Faculty of Health Science, University of La Rioja, Logroño, Spain

**Keywords:** frailty syndrome, chronic disease management, cognitive impairment, personalized interventions, multidisciplinary care, polypharmacy, caregiver support, telemedicine

## Abstract

Frailty syndrome (FS) is a complex and multifaceted condition commonly observed in old adults patients with chronic diseases, often accompanied by cognitive impairments. This review explores the integration of frailty interventions into existing care models to improve patient outcomes, focusing on four key areas. First, it emphasizes the importance of comprehensive assessment tools to identify frailty and cognitive impairments early, facilitating targeted care planning. Second, it highlights the value of personalized interventions, such as dietary modifications, exercise programs, and cognitive training, tailored to individual patient needs and preferences. Third, the review underscores the critical role of multidisciplinary care teams in providing holistic and coordinated care, leveraging the expertise of diverse healthcare professionals. Finally, it examines the potential of technological innovations and caregiver support systems in enhancing frailty management and addressing the challenges posed by cognitive impairments. By integrating these approaches, this review presents a patient-centered framework aimed at mitigating the impact of frailty and improving long-term outcomes. The findings emphasize the need for a unified strategy that combines personalized care, interdisciplinary collaboration, and technological advancements to address the multifaceted challenges of frailty in chronic disease management.

## 1 Introduction

As healthcare systems face the growing burden of chronic diseases, particularly in aging populations, the need for innovative approaches to enhance patient outcomes becomes increasingly urgent (1). Frailty syndrome (FS) is a clinical condition characterized by diminished strength, endurance, and physiological function, resulting in increased vulnerability to adverse health outcomes, particularly in older adults (2). It is a multidimensional concept encompassing physical, cognitive, social, psychological, and nutritional domains, each of which contributes to the complexity of frailty (3). Physical frailty, commonly linked to sarcopenia, manifests as reduced muscle strength and mobility (4). Cognitive frailty combines physical decline with impairments such as memory deficits or diminished executive function (5). Social frailty stems from limited networks and support systems, exacerbating isolation and its associated health risks (6). Psychological frailty includes conditions such as depression or anxiety, which further compound physical and cognitive challenges, while nutritional frailty is characterized by malnutrition or vitamin deficiencies, undermining overall resilience and recovery (7).

FS is closely associated with chronic diseases, such as cardiovascular disease, diabetes, chronic obstructive pulmonary disease (COPD), and osteoporosis (8, 9). Evidence indicates that frailty exacerbates the progression and outcomes of these conditions. For instance, patients with heart failure and frailty experience higher rates of hospitalization and mortality, while individuals with diabetes and frailty often face complications linked to poor glycemic control and comorbidities (7, 10, 11). Epidemiological studies show that frailty affects approximately range between 4.0 and 59.1% of community-dwelling older adults (12), with prevalence increasing to around 50% in those with chronic illnesses (8, 13). Furthermore, the burden of frailty is particularly pronounced in regions with limited access to healthcare and higher rates of multimorbidity (14). Due to global population aging, the prevalence of frailty is projected to rise significantly in the coming decades, posing substantial challenges for healthcare systems worldwide.

Frailty not only diminishes the quality of life for patients but also contributes to increased healthcare utilization and costs due to frequent hospitalizations, prolonged recovery periods, and higher rates of morbidity (15).

Integrating frailty interventions into existing care models offers a promising pathway to address these challenges. Frailty interventions, including targeted assessments, physical rehabilitation, nutritional support, and coordinated care strategies, have the potential to improve patient resilience, reduce the progression of chronic conditions, and enhance overall health outcomes. By embedding these interventions into the fabric of current healthcare delivery systems, it is possible to create a more holistic, patient-centered approach that proactively manages both chronic diseases and their associated risks (16). This paper aims to explore the comprehensive integration of frailty interventions into established care models, examining the evidence supporting their efficacy, the challenges of implementation, and the potential benefits for patients with chronic diseases. In doing so, it highlights the importance of a multidisciplinary approach, where clinicians, caregivers, and healthcare systems work in concert to deliver personalized, preventative care that enhances patient quality of life and reduces the strain on healthcare resources.

## 2 Methods

The search database was PubMed. The retrieval time node ranged from January 2014 to December 2024. The retrieval strategy was optimized with the use of Boolean logical operators. The search terms included a combination of MeSH terms and keywords, such as: “old adults frailty,” “frailty in older adults,” “frail old adults populations,” “frailty and heart failure,” “frailty and multimorbidity,” “multimorbidity and heart failure,” “frailty and cardiovascular diseases,” “frailty and diabetes,” and “frailty and cognitive impairment.” This comprehensive search strategy ensured that the review covered both historical and recent studies, offering a full spectrum of evidence on frailty and its patients with chronic diseases.

All retrieved citations were imported into Zotero for reference management and deduplication. The inclusion criteria were defined as follows: (1) papers covered a population of adults; (2) the paper’s main topic was frailty and frality in the context of chronic diseases (e.g., multimorbidity, heart failure, diabetes, or cognitive impairment); (3) the full text was accessible; and (4) the paper was published in English.

### 2.1 Challenges in interventions for patients with cognitive impairments

#### 2.1.1 Understanding and communication

Interventions designed to mitigate the effects of FS include surgical options (e.g., ventricular assist devices, heart transplantation) and non-surgical options (e.g., exercise and nutritional interventions) (17). Non-surgical interventions can be complex, however. As people with HF and FS often have co-occurring cognitive impairment, non-surgical interventions may be challenging (18, 19). For example, exercise interventions include a multitude of activities designed to improve balance, strength and mobility (1, 4). Clients with HF and FS may have difficulty recalling the exercises and the frequency at which they should be performed due to memory deficits (18, 20). Dietary restrictions focus on increased intake of macronutrients such as proteins and micronutrients such as vitamin D and iron (17, 21). Clients with cognitive impairment may fail to recall nutritional recommendations or understand the association between the nutritional recommendations and improved management of HF and FS (18, 20). Furthermore, as comorbid illness is common in HF, people might have to balance several nutritional recommendations simultaneously (e.g., low-salt and diabetic diet) (17, 18, 20). People with cognitive impairment may have difficulty coordinating multiple nutritional recommendations. Clinicians must be vigilant with frail HF clients that demonstrate cognitive impairment to ensure maximum benefit from non-surgical interventions.

#### 2.1.2 Self-management and monitoring challenges

Cognitive impairments range from mild cognitive decline to severe dementia, and can be classified into subjective cognitive decline (SCD), mild cognitive impairment (MCI), and dementia (22). SCD involves perceived memory issues without measurable deficits, while MCI presents detectable cognitive impairment but allows for some level of independence. In dementia, self-care abilities are typically lost entirely, and patients become dependent on external support for basic daily activities. Cognitive impairments are linked to various negative health outcomes, including poor disease management, increased hospitalization rates, a higher risk of adverse events, and reduced quality of life (23).

Cognitive impairment often accompanies chronic conditions, and act as a “hidden disability” which complicates treatment and patient care (24). Certain chronic illnesses such as cerebrovascular disorders, malignancies, metabolic diseases like diabetes are linked to higher risks of cognitive decline (25). Elderly individuals are at greater risk for cognitive impairments, which frequently coexist with chronic conditions, further complicating the management of their illnesses (26). For example, in heart failure patients, cognitive decline increases the risk of hospitalization and adverse events, while in cases of chronic pain, adherence to pain management regimens becomes more challenging (27, 28).

Self-care plays a pivotal role in managing chronic conditions and maintain overall health. It refers to the actions individuals take to maintain health, monitor illness, and manage ongoing medical conditions Effective self-care leads to better outcomes, fewer hospitalizations, and improved quality of life (29). However, cognitive impairments significantly reduce self-care capacity, particularly in dementia, where memory lapses, symptom misrecognition, and difficulty following health regimens lead to worsened outcomes (30, 31). Self-care requires intact cognitive functions, such as the ability to learn, perceive, and interpret symptoms—skills that are often compromised in patients with cognitive impairments. Without these abilities, patients struggle to recognize symptoms, follow treatment plans, or make timely health decisions (32).

Patients with cognitive impairments face distinct challenges in self-care based on the severity of their condition. For example, in individuals with type 2 diabetes, cognitive impairment increases the risk of non-adherence to prescribed therapies, leading to poor disease control and preventable complications (33). Although patients outwardly struggle with managing their chronic illnesses, the underlying cognitive impairment often goes unnoticed (34). This hidden aspect complicates treatment, as patients may fail to adhere to medication schedules, manage dietary restrictions, or attend follow-up appointments, not due to a lack of effort, but because of unrecognized cognitive limitations (35). Even in early-stage dementia, self-care challenges such as medication adherence and hygiene management remain significant (36). Moreover, cognitive impairments undermine critical aspects of self-care, including the ability to learn new information and interpret symptoms, thereby complicating the management of complex chronic conditions (37).

Addressing the challenges of self-care in cognitively impaired patients is complex due to overlapping factors. Traditional assessment tools, such as neuroimaging and cognitive tests, measure cognitive decline but do not effectively capture self-care capacity. Self-report tools are often unreliable due to memory deficits. Digital tools, including online cognitive training programs, offer a valuable complement to traditional methods, providing real-time, more accurate assessments of cognitive function and self-care abilities (36).

Several interventions have been found effective in improving self-care in cognitively impaired patients. Cognitive training programs, for instance, have been shown to help patients with MCI maintain self-care abilities. More severe impairments, such as dementia, necessitate caregiver-supported care to ensure that daily tasks and health decisions are effectively managed (38). Despite progress, there is still a gap in fully understanding self-care behaviors in patients with cognitive impairments, which highlights the need for further research. Technologies, such as mobile apps and wearables, offer potential support for patients by assisting with medication reminders and other self-management tasks (38).

Self-efficacy, or the belief in one’s ability to manage health, plays a critical role as a mediator of self-care behaviors. Patients with higher self-efficacy are more likely to engage in effective self-care practice (39, 40). Programs focusing on memory and attention exercises have been shown to improve self-care behaviors in patients with mild cognitive impairment (41). In this regard, caregiver-supported interventions are particularly valuable, as caregivers can provide the structure needed to improve adherence to treatment regimens (42). Additionally, patients with strong social support networks tend to experience better self-care outcomes, while those living alone are at higher risk for poor disease management and faster cognitive decline (26).

As cognitive impairment progresses, patients require increasing levels of supervision (43). Caregivers, whether family members or professional healthcare workers, play a crucial role in ensuring that these patients receive appropriate care, from administering medications to assisting with daily tasks (44). Constant supervision improves safety and helps prevent dangerous situations such as falls, medication errors, or malnutrition. However, the burden on caregivers can be significant, often resulting in caregiver burnout, which affects the quality of care and the overall health of both the patient and caregiver (45). Increased support from healthcare providers is crucial to help alleviate this burden and ensure the safety and well-being of both parties (46).

Denial of cognitive decline poses a significant barrier to timely intervention, particularly in early dementia (47). This denial delays diagnosis and reduces the effectiveness of interventions that could slow disease progression and deteriorate quality of life (48). Furthermore, social isolation can exacerbate both cognitive decline and physical health issues. The absence of regular social interaction often accelerates the progression of conditions like dementia, while also increasing depression and reducing physical activity levels (49, 50). This cycle of social withdrawal and declining health highlights the importance of integrated interventions that address both the social and cognitive needs of patients, as well as early detection and ongoing support from healthcare providers. Cognitive impairment greatly hinders effective self-care in chronic conditions, often leading to poor health outcomes. Early detection, tailored interventions, and constant caregiver support are essential for improving patient care and quality of life.

Memory and Task Execution Problem. The prevalence of cognitive decline, particularly dementia, increases exponentially with advancing age. Cognitive impairment occurs in over 40% of older people (51). FS is very common in people with dementia (50.8 to 91.8%). People with FS use polypharmacy much more often (52). Older people often have chronic diseases that require a strict therapeutic regimen (e.g., arterial hypertension). The level of adherence to antihypertensive treatment, depending on age, takes the shape of a U curve. The most adherent to the therapy are patients around 65 years of age, while the lowest level of adherence to therapeutic recommendations is observed in patients aged 30 and > 80 years (53). Cognitive impairment significantly affects adherence to therapeutic recommendations. A study of 436 older people showed that 48.6% of them had poor medication compliance. The presence of cognitive impairment increased the risk of medication non-adherence by almost 3-fold (OR = 3.95; 95% CI: 2.63–5.92, *p* < 0.001) (54). High medication concerns among frail older patients inhibit their medication adherence (52). In older people using polypharmacy, a number of adverse situations related to treatment may occur: (1) neglecting to fill a prescription for a recommended medicine; (2) skipping at least one dose; (3) taking the wrong medication; (4) taking an excessive amount of a prescribed medication; (5) stopping a medication too soon; (6) incorrect use of medical equipment like inhalers and syringes, and (7) taking damaged, expired, or improperly stored medicines. All of this makes older people require more detailed instructions for using medications and educational and behavioral interventions that improve adherence to treatment (55). Self-management interventions and electronic health interventions might be effective in improving medication adherence for older people with multimorbidity (56). Cognitive-based behavior change techniques are effective interventions eliciting improvements in medication adherence that are likely to be greater than the behavioral and educational interventions largely used in current practice (57). Whenever possible, combined treatment should be recommended, e.g., in patients with hypertension and lipid disorders (one tablet containing two antihypertensive drugs and one tablet containing a statin and ezetimibe - this allows for a 50% reduction in the number of tablets). The use of combined treatment improves adherence and is associated with a lower risk of adverse effects (58). In people with cognitive disorders and FS, cognitive training is very beneficial. It has been shown that cognitive training significantly benefits overall cognitive function, delayed memory, orientation, attention, and language skills in aged patients with cognitive impairment (59). This may significantly translate into a greater likelihood of compliance with medical recommendations regarding the treatment of chronic diseases. Increasing the level of adherence is very important because it has been shown that good adherence was associated with a 21% reduction in long-term mortality risk in comparison to medication non-adherence (adjusted hazard ratio 0.79, 95% CI 0.63, 0.98) (54). In old adults patients with polypharmacy, the medications used should be assessed as well as the patient. It is recommended that in the context of the medications used: (1) assess drug–drug, drug-disease interactions; (2) identify non-beneficial therapy and simplify; and (3) identify high risk therapy and reconsider, while in the context of the patient assessment, it is recommended to: (1) medication reconciliation (are all medications indicated?); (2) assess adherence and each barrier; (3) adjust for elimination avoiding toxicity (clearance, metabolism); (4) identify functional status, deficits and readdress medication resources; and (5) identify goals of care and adjust for medications that are consistent (60).

### 2.2 Selected chronic diseases and frailty

Hafızoğlu et al. recently presented the results of an interesting study in which they analyzed which indicator related to the measure of multimorbidity is most appropriate in the context of frailty. The relationships between four multimorbidity indices (CIRS-G, ACCI, GIC, ICED) and three scales related to the severity of frailty (FRAIL, CFS, TFI) were analyzed. CIRS-G was found to be the most appropriate indicator (61). The CIRS-G scale includes an assessment of the following systems or organs for chronic diseases: cardiovascular system, circulatory system, respiratory system, vision, hearing, pharynx and larynx, digestive system, excretory system, musculoskeletal system, nervous system and endocrine system (62). It is therefore worth paying attention to the relationship between selected most common chronic diseases from the above-mentioned areas and frailty syndrome.

Recently, results from a study were presented that evaluated the impact of a 12-week nursing intervention based on the integration theory of health behavior change in frail older adults with type 2 diabetes mellitus. Among other things, significant improvement was observed in frailty level (*p* = 0.006) and quality of life (all *p* < 0.001) (63). Similarly, according to a recently published systematic review, the exercise, diet, and education programs were shown to reduce the risk of frailty or progression to more advanced stages in patients with type 2 diabetes and frailty (64).

The relationship between frailty syndrome and arterial hypertension is worse. Both hypertension increases the risk of frailty and frailty syndrome has a negative impact on hypertension management (65). Nevertheless, it remains questionable to what extent antihypertensive treatment has a beneficial effect on frailty status in this population (66).

According to recently published results, not only overt cardiovascular disease, but even signs of subclinical dysfunction or damage, such as increased levels of natriuretic peptides or cardiac troponin, translate into a significant increase in the risk of frailty syndrome (67). According to a study by Zhong et al., different cardiovascular therapeutic interventions may have different effects depending on frailty status (68). Ijaz et al. drew attention to the multitude of interventions to improve the frailty status in patients with cardiovascular diseases (classified into physical, pharmacological, cognitive, nutritional, and psychosocial interventions) and the need to individualize the management depending on the needs of a specific patient (69).

Pulmonary rehabilitation has been shown to have a positive effect on handgrip strength in patients with COPD. Interestingly, positive handgrip strength delta was associated with higher baseline quality of life scores (70). Importantly, exercise intervention may also have a positive impact in frail patients hospitalized due to COPD exacerbation as an adjunct to standard treatment (71).

In a study by Yamashita et al., it was found that proactive foot care can be of significant importance in reducing the risk of fractures in frail older individuals (72). This is particularly important for patients at increased risk of fractures, such as those with osteoporosis.

### 2.3 Nutrition and physical activity

#### 2.3.1 Nutritional status assessment

Poor nutritional status is regarded as one of the key, modifiable clinical markers of FS. Comprehensive nutritional assessment plays an essential role in the holistic approach to chronic disease management, aiming to improve patient outcomes and quality of life (73). Malnutrition is associated with FS, and screening and early identification of malnutrition can help prevent the progression of disability, particularly among older adults (74). Tools for assessing malnutrition include body mass index (BMI), anthropometric measures, biochemical markers, and nutritional risk assessment scales (75). While BMI is widely used, it may be unreliable due to confounding factors such as edema or lack of specificity regarding body composition. Furthermore, when used as a single marker, BMI may fail to capture the phenotypic presentation of FS, often characterized by unintentional weight loss exceeding 4.5 kg or ≥ 5% (76).

For malnutrition risk assessment, tools like the Mini Nutritional Assessment (MNA) and Nutritional Risk Score 2002 (NRS-2002) are available. The European Society for Clinical Nutrition and Metabolism (ESPEN) recommends the standardized criteria of the Global Leadership Initiative on Malnutrition (GLIM), involving a two-step procedure: initial screening with any validated tool, e.g., NRS-2002, SGA, or MNA, followed by comprehensive diagnostics including phenotypic and etiologic criteria along with the assessment of malnutrition severity (77). In comprehensive assessment, both quantitative and qualitative parameters should be considered. Many studies describe an inverse relationship between protein intake and the prevalence of FS, although evidence remains heterogeneous. Sarcopenia, associated with the loss of muscle mass and strength, is considered a critical component of frailty and appears linked to protein intake (78, 79). Not only daily protein intake but also its distribution throughout the day impacts the incidence of FS (74).

In a meta-analysis by Coelho et al., high dietary protein intake was inversely correlated with frailty status in older adults (80). In a subsequent study by the same author, although protein intake was not significantly associated with frailty in older adults, it was noted that protein sources could play a key role in the development of frailty, with higher intake associated with lower FS risk (80). Thus, assessing protein intake quality by a qualified dietitian, along with identifying potential deficiencies, can aid in identifying patients at risk. A 24-h dietary recall or a nutritional interview may assist in evaluating intake.

Individuals with FS generally exhibit lower intake of energy, protein, fiber, vitamin D, vitamin C, folate, and B vitamins compared to non-FS individuals. Thus, screening for potential deficiencies is a key component. Vitamin D deficiency, for example, is associated with poorer physical function and may predict physical disability in older adults (81). The relationship between vitamin D levels and frailty has been evaluated in numerous studies. Observational data from Hirani et al. indicated that low vitamin D levels are independently associated with frailty (82). Wang et al. confirmed that lower levels of vitamin D (RR: −3.22, 95% CI: −3.86 to 2.59, *p* < 0.001) may be associated with frailty. Notably, vitamin D is essential for maintaining calcium homeostasis and bone health, as low calcium levels may contribute to FS through mechanisms affecting muscle strength and bone density. Additionally, prevalent osteoporosis and sarcopenia in older adults increase susceptibility to frailty, disability, hospitalization, and decreased quality of life (83). Similar deficiencies may include other vitamins, such as vitamin B6 and folate, as well as trace elements and minerals (84, 85). A cross-sectional study among women aged 70–80 participating in the Women’s Health and Aging Studies I and II demonstrated a higher likelihood of low trace element levels among FS patients (86). Therefore, conducting a thorough nutritional analysis and managing FS-specific nutrient deficiencies provide a multifaceted approach and should be implemented in this patient group.

#### 2.3.2 Nutritional interventions

Multimodal interventions, including effective nutritional strategies, should aim to mitigate adverse health effects and reduce FS severity (87). Nutritional support should be personalized, with nutrient intake adjusted based on a thorough analysis of nutritional status, physical activity level, and patient condition.

A key component of nutritional intervention is meeting the patient’s energy requirements. Low energy intake commonly affects older individuals, particularly between the fourth and seventh decades of life (84, 88). A study by Kim et al. demonstrated that protein-energy supplementation among old adults patients with low socioeconomic status slows functional decline (89). Therefore, exploring interventions to stimulate food intake is crucial. This could include strategies such as meal fortification, additional snacks, dietary fortification (particularly with oral nutritional supplements, ONS), enhancing palatability, ensuring high energy density, and providing suitable meal forms (88). ONS can be beneficial for improving nutritional status, especially when combined with multifactorial interventions (89, 90). Proper identification and treatment of unintentional weight loss and malnutrition are recommended among FS patients (91).

Among older adults, protein intake may be insufficient, leading to reduced physical performance and poorer clinical outcomes. Evidence supports the effect of protein supplementation on clinical outcomes. Although the optimal protein intake for older adults remains undefined, a study by Vellas et al. found that women consuming over 1.2 g/kg body weight/day of protein experienced fewer health issues compared to those consuming 0.8 g/kg/day (84, 92). In research by Bonnefoy et al., protein-energy supplementation did not significantly increase lean body mass but improved muscle strength in older FS patients (93). Protein intake for older adults should be tailored to nutritional status, physical activity level, health condition, and tolerance, ranging from 1.0–1.2 g/kg body weight in healthy individuals to up to 2 g/kg in severely malnourished or acutely ill patients (94, 95). Another potential intervention includes even distribution of protein across meals. Loenneke et al. demonstrated that consuming meals with 30–45 g of protein per meal was associated with greater strength and leg lean mass (96). Though further research is needed, even protein distribution throughout the day may enhance not only patient outcomes but also total daily protein intake. Additional studies on the quantity, quality, and types of protein intake, as well as new strategies addressing impaired muscle protein synthesis related to the inflammatory state in FS, are needed (84).

There is a significant gap in the literature on the effects of mineral and vitamin supplementation in FS patients, and results from current studies remain inconclusive (97). Some evidence suggests that micronutrient supplementation may improve nutritional status and functional capacity, though its impact on frailty is unclear, as highlighted by a recent systematic review (98). While vitamin D intake is critical, particularly among older adults due to its association with bone health, muscle strength, and function, the impact of supplementation on FS remains uncertain (91). A meta-analysis by Muir et al. found that daily vitamin D supplementation (20–25 μg) was associated with improved gait speed and muscle strength in older adults (99). Furthermore, old adults individuals living with FS may have reduced sun exposure, indicating a need for vitamin D supplementation (100).

**Figure 1:**
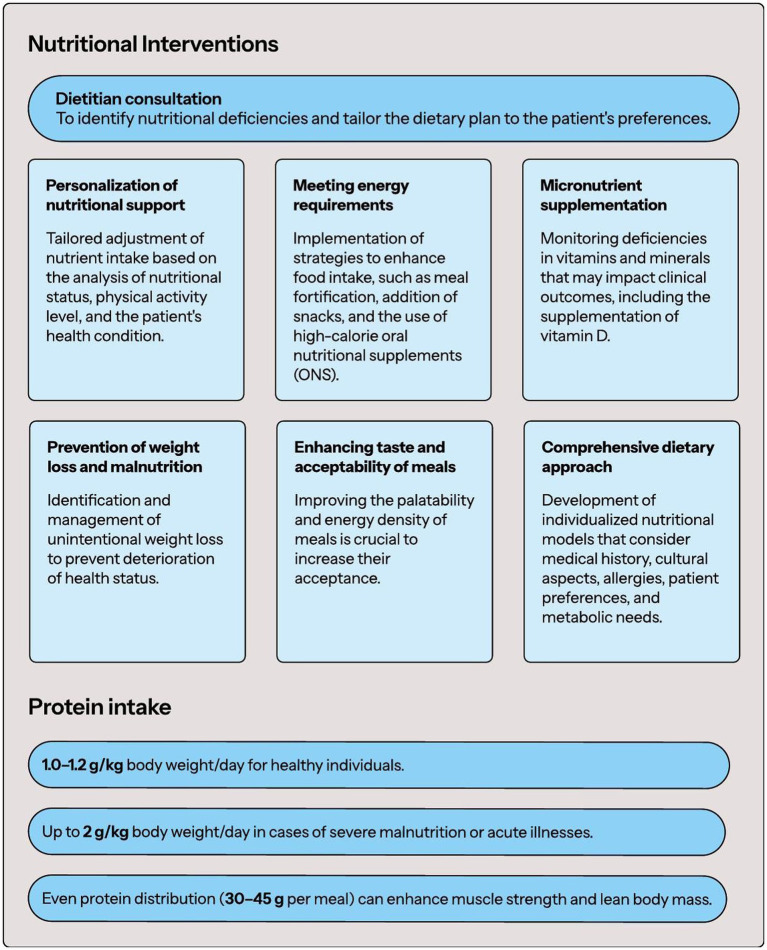
Nutritional interventions in FS.

A critical element in the personalization of dietary intervention is recognizing that a patient’s diet may not meet basic nutrient needs, including minerals and vitamins, and deficiencies may indirectly affect clinical outcomes. Consulting a qualified dietitian and conducting a detailed nutritional assessment may help identify issues and provide appropriate nutritional support. Selecting the right dietary model and nutritional intervention should involve a thorough dietary and health history and be tailored individually to the patient’s condition. The nutrition plan should consider patient preferences, cultural aspects, potential allergies, and the need to address any nutrient deficiencies. Figure 1 provides a summary of dietary interventions used in FS.

#### 2.3.3 Physical exercise programs

**Figure 2:**
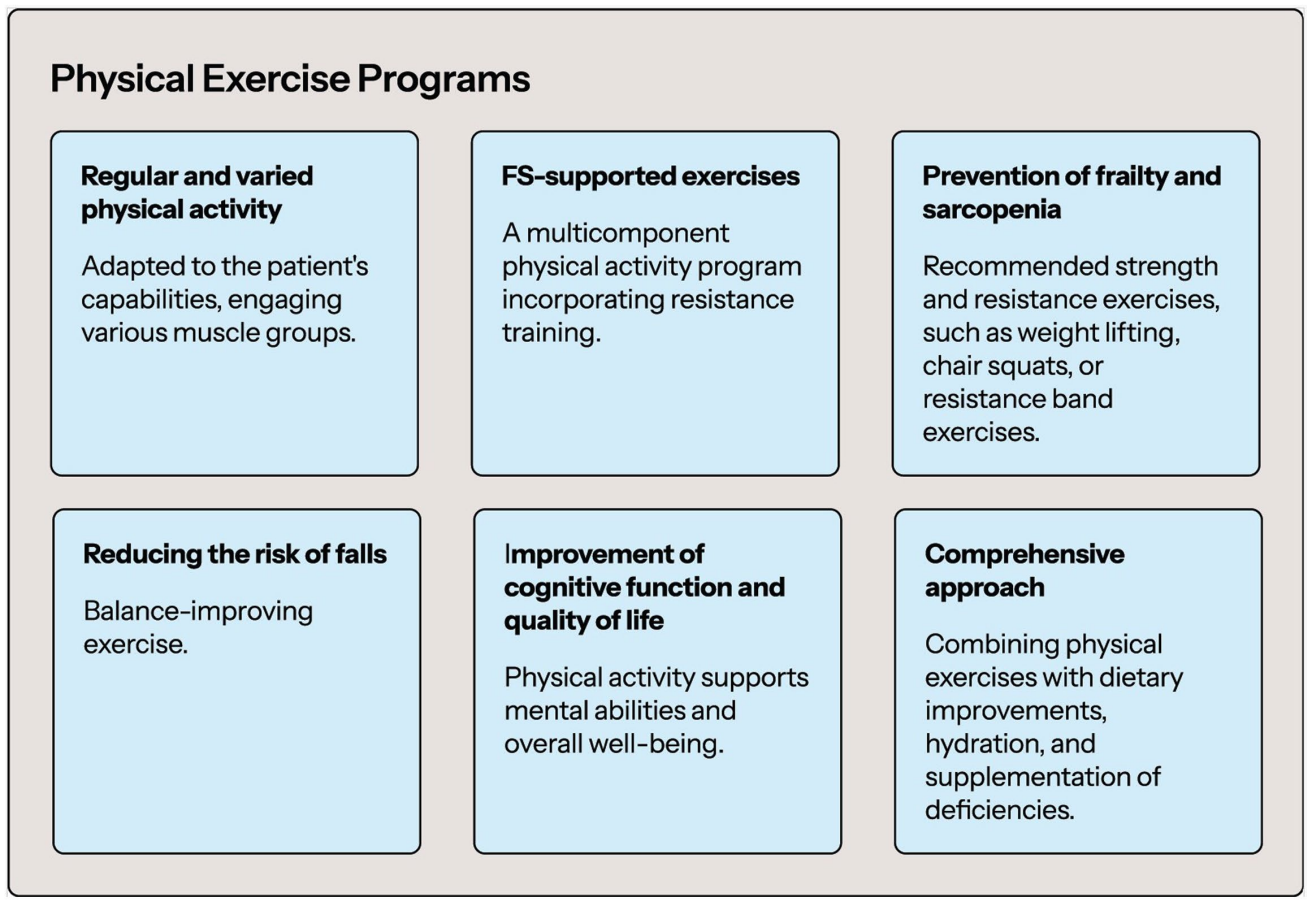
Physical exercise programs summary.

Low levels of physical activity are much more common in people with FS (101). It is worth emphasizing that people with higher levels of physical activity are characterized by a 37% lower risk of developing FS (ES 0.63, 95% CI: 0.52–0.77) (102). Physical activity/exercise is considered one of the main strategies to counteract frailty-related physical impairment in the old adults (103). There are various physical exercise programs available to improve the condition of patients with FS. One of them is the VIVIFRAIL Multicomponent Physical Exercise Program to Prevent Frailty and the Risk of Falls, which includes seven different exercises: (1) walking (when muscle strength allows, otherwise it should be improved first; walking time should be gradually extended depending on individual capabilities); (2) squeeze a ball (12 repetitions, 3 sets); (3) lift a bottle (12 repetitions, 3 sets); (4) extend legs using a ballested ankle brace (12 repetitions, 3 sets); (5) standing up from a chair with the help of a companion (12 repetitions, 3 sets); walking with feet on a line (15 paces, 3 sets); and (6) strange arms (3 repetitions, 3 sets; maintain 10 s). Example of exercise wheels for each functional level that include the exercises, series and repetitions that should be done every week (104). It should be emphasized that the progressive decrease in intrinsic capacity and functional reserve (ultimately leading to disability) can be significantly slowed down at the pre-frailty and frailty stages if appropriate therapeutic intervention is undertaken (a program of appropriate physical exercises is implemented). At the disability stage, the reversibility of the lost intrinsic capacity and functional reserve is much more difficult to achieve (103). The extraordinary importance of physical activity in people with FS is emphasized by the fact that physical activity might partly compensate for the greater mortality risk associated with frailty in old age (105). In the context of implementing a program of appropriate physical exercises, special attention should be paid to people who were less physically active earlier in life. The risk of FS in such people is significantly higher, therefore such people require special education and physical activity programs to reduce this risk (105). Maintaining an appropriate level of physical activity in people with FS is also associated with a significant improvement in global cognition and mental flexibility (106). FS is one of the most important factors increasing the risk of falls in the old adults (107). Physical activity, in addition to its beneficial effect on longevity and cognitive functions, contributes to reducing the risk of falls in frailty patients (risk ratio 0.66; 95% CI: 0.52–0.84) (108). It should be emphasized that physical activity, in order to be as beneficial as possible in people with FS, should be regular, involve different parts of the body and should be adapted to the individual capabilities of the patient. The problem of sarcopenia in patients with FS cannot be forgotten. Sarcopenia and frailty co-occurred in 12.1% of the patients. The co-occurrence of FS and sarcopenia worsens the prognosis of patients (109). It has been shown that physical activity contributes to reducing the risk of both FS and sarcopenia in the old adults (110, 111). Physical activity reduces the risk of sarcopenia in the old adults by up to 50% (110). First-line therapy for the management of frailty should include a multi-component physical activity program with a resistance-based training component (strong recommendation) (112). Of course, it is best if the health intervention in a person with FS includes not only physical activity, but also cognitive exercises, improving eating habits (including proper hydration and supplementation of possible deficiencies) (113). Figure 2 provides a Physical Exercise Programs in FS.

## 3 Role of caregivers and multidisciplinary team

### 3.1 Role of caregivers: caregiver burden and support: challenges associated with caring for patients, and the need for education and support for caregivers

Informal caregivers play a pivotal role in supporting patients with chronic illnesses, ensuring that these individuals adhere to treatment plans and prevent complications (114). Their contribution is particularly important when patients must adhere to complex treatment regimens and require assistance with daily care (29). Involving caregivers in daily self-care tasks is critical to preventing poor health outcomes and improving the patient’s overall well-being. Active caregiver involvement has been shown to significantly reduce symptom burden and enhance chronic illness management (115).

The role of caregivers becomes even more important when cognitive impairment, such as dementia, coexist with the chronic illness. In these cases, caregivers must deal not only with the physical demands of care, but also with the difficulties determined by cognitive dysfunctions, such as memory lapses, and confusion, which create, above others, significant challenges in adhering to the treatment plan (116). There is evidence that caregivers of patients with dementia are often more stressed than those caring for other illnesses and face greater emotional and mental distress, which can lead to higher rates of depression and anxiety (117).

Caregiving preparedness is another key factor in ensuring effective care. Preparedness refers to the caregiver’s perceived ability to handle the emotional and physical responsibilities of caregiving. Studies have shown that caregivers who are better prepared for their roles suffer less from anxiety and depression, which enhances their ability to provide consistent, high-quality care (118). In addition, caregiver confidence, the belief in their own ability to provide effective care, is critical. Confident caregivers are more likely to contribute positively to patient self-care, maintain patient stability, and respond appropriately to health deterioration (118). Therefore, promoting caregiver preparedness and confidence is critical to improving both patient and caregiver outcomes.

Despite the positive aspects the caregiving experience entails (e.g., personal growth and role satisfaction) (119), caregivers face numerous challenges, particularly when caring for patients with both chronic illnesses and cognitive impairment (120). Physical strain is common, as tasks such as assisting with mobility and personal care can lead to chronic pain and fatigue (121). Emotionally, caregivers experience increased stress due to the psychological and behavioral symptoms associated with conditions such as dementia (122). Over time, these stressors can lead to burnout, which not only affects the well-being of the caregiver but also diminishes the quality of care provided to the patient (45).

Other factors that hinder the caregiving process include social isolation, loneliness, and financial burden. Due to the demanding nature of their responsibilities and tasks, caregivers often become isolated and without social support (123). This lack of connection exacerbates emotional fatigue and contributes to a decline in mental health. Additionally, financial burden is common, as many caregivers reduce their working hours or quit their job entirely to provide care (124). These cumulative challenges highlight the need for comprehensive support systems to reduce caregiver burden.

To improve the health outcomes of both members of the dyad, enhancing caregiver contribution and preparedness is essential, particularly for patients with cognitive impairment. Educational programs (i.e., courses on medication management, emotional support initiatives, and digital platforms such as mobile apps and virtual reality) have proven effective in improving caregiver outcomes (125), especially those that equip caregivers with the skills needed to manage medical tasks and symptoms. Respite care is another strategy that could be implemented because it offers temporary relief from their duties and reduces the risk of burnout (126). Access to mental health services and support groups is equally important, providing emotional validation and helping caregivers cope with the stresses of their role (127, 128). For caregivers who are alone, tailored interventions, such as mindful meditation, computer-based applications, and music therapy, are essential to help reduce feelings of isolation and loneliness. These interventions not only provide emotional relief but also enhance caregivers’ ability to cope with the stress (128). Caregivers are essential for managing chronic illnesses, particularly when cognitive impairment is present. Improving caregiver preparedness and providing comprehensive support systems are essential to ensuring high-quality care for patients while safeguarding the caregiver well-being.

### 3.2 Multidisciplinary team

An old adults person with FS is characterized by very complex problems that significantly worsen their quality of life. A meta-analysis covering over 54 thousand old adults people showed that frailty and pre-frailty, according to a multidimensional definition, are common in older people affecting, respectively, one person over four and one over three (129). Frailty, geriatric syndromes, disability and multimorbidity often overlap and increase the risk of mortality and poorer quality of life (130). It is worth emphasizing that the prevalence of multimorbidity in frail individuals was 72% (131). FS covers several domains of human functioning: (1) Physical condition: sarcopenia, physical insufficiency, oral dysfunction, malnutrition; (2) Mental condition: cognitive impairment, dementia, depression; (3) Social condition: social isolation, living alone, lack of social support, economic deprivation (132). This indicates the need for comprehensive, multi-specialist and holistic care for these patients. Optimal care for a patient with FS should be multidirectional and include: (1) improving eating habits, supplementing deficiencies, proper hydration (an important role for a clinical dietitian); (2) broad education regarding, among others, the need to follow therapeutic recommendations (an important role for a nurse); (3) improving physical condition and implementing a program of individually adapted physical exercises (an important role for a physiotherapist-rehabilitator); (4) assessing the need for and monitoring the medications used (an important role for a doctor and pharmacist); (5) controlling comorbidities (an important role for doctors of various specialties); (6) assessing health needs (an important role for a geriatrician) and (7) psychological support (an important role for a psychologist) (133). The following scales are extremely helpful in assessing the severity of the FS and specifying individual needs: (1) Multidimentional frailty assessment: Frailty Index of Cumulative Deficits; Edmonton Frail Scale; Tilburg Frailty Indicator; Groningen Frailty Indicator; Kihon Checlist; (2) Cognitive impairment assessment: Mini-Mental State Examination; Montreal Cognitive Assessment; Mini-Cog; Short Portable Mental Status Questionnaire; (3) Physical Frailty Assessment: Fried’s Frailty Phenotype; FRAIL Scale; Frailty Screening Index; Clinical Frailty Scale; Short Physical Performance Battery; (4) Social frailty assessment: Makizako’s Questionnaire; Garre-Olmo’s Questionnaire; Teo’s Questionnaire; Lubben Social Network (132). An extremely important aspect is to increase the awareness of medical personnel and families of older people with FS about their individual health needs. Table 1 presents a summary of comprehensive, multi-specialist, and holistic care for frail patients.

**Table 1:** Summary of comprehensive care for the frail patient (132).

Comprehensive, multi-specialist and holistic care for frailty patients	
Task	Key person
Improving eating habits, supplementing deficiencies, proper hydration	Clinical dietitian
Broad education regarding, among others, the need to follow therapeutic recommendations	Nurse
Improving physical condition and implementing a program of individually adapted physical exercises	Physiotherapist-rehabilitator
Assessing the need for and monitoring the medications used	Doctor and pharmacist
Controlling comorbidities	Doctors of various specialties
Assessing health needs	Geriatrician
Psychological support	Psychologist
Helpful tools for assessing patients with frailty syndrome	
Multidimentional frailty assessment: Frailty Index of Cumulative Deficits; Edmonton Frail Scale; Tilburg Frailty Indicator; Groningen Frailty Indicator; Kihon Checlist; Cognitive impairment assessment: Mini-Mental State Examination; Montreal Cognitive Assessment; Mini-Cog; Short Portable Mental Status Questionnaire; Physical Frailty Assessment: Fried’s Frailty Phenotype; FRAIL Scale; Frailty Screening Index; Clinical Frailty Scale; Short Physical Performance Battery; Social frailty assessment: Makizako’s Questionnaire; Garre-Olmo’s Questionnaire; Teo’s Questionnaire; Lubben Social Network.	

## 4 Medication management and safety

### 4.1 Medication management

Polypharmacy is one of the critical issues that is discussed in the context of FS, especially in the old adults. There is no one commonly accepted definition of polypharmacy. According to the report prepared by the World Health Organization, polypharmacy can be defined as “the concurrent use of multiple medications” and it is “often defined as the routine use of five or more medications” (134). Definitions available in the literature are differentiated. Some definitions include only a number of drugs, or in combination with the duration of therapy, or even in a health care setting (135). Although polypharmacy can be partially considered as a consequence of multimorbidity (136) and, therefore, difficult to avoid in order to treat simultaneously multiple chronic diseases in accordance with current medical knowledge, the literature discusses it as a significant problem, mainly in the old adults, and a risk factor that can lead to further decreases in health state (137, 138). There is no doubt that the list of medications used by a patient must be regularly verified to avoid inappropriate prescribing (139). The risk of iatrogenic complications increases especially in patients with FS (140), who are characterized by reduced physiological reserves and reduced resistance to stress factors (141).

Gutiérrez-Valencia presented the results of a systematic-review including 25 observational studies. Most publications included in the analysis (16 of 18 cross-sectional studies and five of seven longitudinal analyses) indicate a significant association between polypharmacy and frailty (142). It is worth noting that different conclusions from individual studies may result from different definitions of polypharmacy. Some studies indicate that the use of six or more medications may be considered a good predictor of an increased risk of developing FS (143, 144).

Polypharmacy was shown to increase risk of frailty in different populations.

A strong positive correlation was found between frailty status (assessed by Clinical Frailty Scale) and polypharmacy (defined as taking at least five drugs) in 298 primary care patients aged at least 65 years (145). Similarly, a significant association between polypharmacy and risk of frailty was shown on a group of 124 hospitalized patients (146). Hemodialysis patients taking more than 11 drugs has significantly higher risk for frailty occurrence at baseline than patients taking fewer than eight drugs (OR 1.54, 95% CI 1.05–2.26). Moreover, the risk for frailty development in two-year observation is significantly higher in patients taking more than 11 medications than patients taking fewer than eight drugs (sub-distribution HR 2.15, 95% CI 1.32–3.48) (147). Significant impact of number of medications on frailty occurrence was shown also in other study involving 388 Japanese hemodialysis patients (OR = 1.351, 95% CI 1.163–1.570) (148). In the group of patients with HIV infection, each additional chronic non-antiretroviral medication is associated with a 4% increase in risk of having an adapted frailty-related phenotype domain (OR 1.04; 95% CI 1.03, 1.05) (149). According to another study, in older patients with HIV infection analogous value of increase in the risk of frailty diagnosis was assessed at the level of 6% (adjusted OR 1.06, 95% CI 0.002–0.11, *p* = 0.04) (150). In a retrospective study including older critical care patients, the patient median Clinical Frailty Scale was shown to increase by 1 with polypharmacy classification increments (*p* < 0.001) (151). Taking at least eight medications was found to be significantly associated with frailty in patients with blood neoplasms (adjusted OR 2.82, 95% CI 1.92–4.17) (152). Pre-frailty and frailty was associated with increased risk of polypharmacy also in patients with history of cancer (OR 8.26, 95% CI 1.74–39.2) (153). Polypharmacy is associated not only with frailty but also pre-frailty. In an observational, cross-sectional study in which 201 patients participated, individuals with frailty and pre-frailty were shown to have increased risk for polypharmacy when compared to patients with no features of frailty (OR 2.36, CI 95% 1.05–5.37; *p* = 0.04) (154). In community dwelling older adults in Europe, a prevalence of polypharmacy was found to be three times more prevalent in frail patients and two times in pre-frail individuals, when compared with patients without features of FS (155).

According to a meta-analysis prepared by Wang et al., polypharmacy was shown to be a significant risk factor for FS development (RR 1.72, 95% CI 1.17–2.28, *p* < 0.001 in studies taking into consideration a precise number of drugs; RR 1.49, 95% CI 1.39–1.60, *p* < 0.001 in studies not taking into consideration a precise number of drugs). Interestingly, in the same meta-analysis such factors as age, stroke, and cardiac diseases, were not shown to be significantly associated with increased risk of frailty (156). In a meta-analysis of 13 studies it was found that patients with frailty and heart failure are characterized by a higher risk of polypharmacy (OR 1.87, 95% CI 1.72–2.04, *p* < 0.01) compared to patients without frailty (157). Similarly, a meta-analysis of 12 studies shown polypharmacy to be a risk factor for frailty in patients with chronic obstructive pulmonary disease (158).

There are also data showing that polypharmacy may also be associated with worse outcomes. A large analysis conducted using the Korean National Health Insurance claims follow-up data from 2002 to 2017 on a group of 55,228 people found that polypharmacy was associated with increased risk for all-cause mortality, hospitalization, and emergency room visits among older colorectal cancer survivors (159). Similarly, polypharmacy is associated with worse prognosis in patients with severe aortic stenosis (160). Interestingly, de Breij et al. found polypharmacy to be an explanatory factor of the association between frailty and mortality in the old adults (161).

Taking into consideration significant impact of polypharmacy to frailty development, it can be expected that deprescribing could be beneficial in patients predisposed to FS. Deprescribing could be defined as the process of medication withdrawal or dose reduction to correct or prevent medication-related complications (162). Ibrahim et al. presented the results of a systematic review of studies aimed to assess the impact of deprescribing among old adults with frailty. Although the authors emphasized that there is a paucity of research in this area, included studies suggest that deprescribing can lead to important benefits (163). Dinarvand et al. described the critical role of the cooperation of geriatricians and pharmacists in deprescribing in the frail ageing population treated due to diabetes and hypertension. Moreover, necessity for further trials has been emphasized (164).

## 5 Patient safety

In the case of people with FS, ensuring the patient’s safety by avoiding factors that may predispose to adverse events is particularly important. In the context of pharmacotherapy, it is crucial to avoid drugs that may contribute to reduced skeletal muscle tone and excessive sleepiness what is associated with increased risk of falls. Medicament with anticholinergic properties were shown to be independently associated with increased of falls in frail (adjusted OR = 3.84, 95% CI 1.48–9.93, *p* = 0.006) and pre-frail participants (OR = 2.71, 95% CI 1.25–5.89, *p* = 0.012), but not in robust subjects (165). According to the analysis based on data from the National Health Interview and Examination Survey for Adults 2008–2011, use of psychotropic drugs is independently associated with an increased risk of falls (OR 1.64, 95% CI 1.14–2.37), especially for selective serotonin reuptake inhibitors (OR 6.22, 95% CI 2.28–17.0) (166).

In the case of old adults people with frailty, it may be necessary to modify the pharmacological treatment of chronic diseases, especially in the field of drugs affecting blood pressure and antidiabetic drugs. The proportion between the expected benefits of excellent control of chronic diseases and the risk associated with pharmacotherapy should be considered in the context of life expectancy. In many cases, it is beneficial to adopt less restrictive therapeutic goals for the control of chronic diseases to avoid complications of possible hypotension and hypoglycemia what also can lead to increased risk of falls as well as decline in cognitive function (167). Therefore, in the case of old adults people with severe FS (at least 5 points on the Clinical Frailty Scale), a satisfactory level of diabetes control can be considered as HbA1c < 8.5%. If it is necessary to reduce the amount of medication used, it is worth considering first discontinuing insulin and sulfonylurea derivatives, i.e., drugs that can lead to hypoglycemia (168). In a large study by Nguyen et al. (11,400 participants, including 25.7% frail), it was confirmed that intensive glucose-lowering and antihypertensive treatments may be less beneficial in patients with frailty (169). However, it should be emphasized that the research results regarding the relationship between chronic disease control and the risk–benefit balance in frail patients are not fully unambiguous. O’Donoghue et al. emphasized that different frailty classifications may be associated with different prognostic implications for the purpose of the application of hypertension management guidelines (170). On the other hand, according to Wang et al. patients with frailty should be treated similarly to other patients, because there is no significant difference in benefits from intensive blood pressure control without an increased risk of serious adverse events (171).

## 6 Tailoring interventions to individual needs

### 6.1 Personalization of interventions

Personalization of interventions is now universally recognized as a key aspect in the treatment of frailty (172). These come primarily through personalized care planning (PCP), which is considered in effect an intervention with which known positive outcomes are associated (173). In fact, PCP is associated to a greater control of the own health and to an improvement of physical and mental health (173) and, in particular, to an increased willingness to adhere to healthy behaviors (174). As described above, frailty has a multidimensional nature. In this perspective, a multi-domain assessment, leaded by a multidisciplinary team, is mandatory and should be the first step in order to counteract frailty (175). In addition to the pharmacological approach, it is well known in the literature that exercise, nutritional interventions and cognitive support represent effective approaches in treating frailty, even being able to reverse its course toward healthy and active aging (176). The following chapters will elaborate on these types of interventions.

### 6.2 Tailored exercise programs: adapting exercise programs to the individual capabilities of the patient

Recent systematic reviews with meta-analysis agree that exercise brings benefits in counteracting frailty (102, 177). It remains unclear what type of exercise is optimal for the frail old adults, although variation of interventions seems to bring benefits (177). However, the first step should be an in-depth assessment of the persons’ abilities and capabilities. This assessment should include, but should not be limited to: skeletal muscle, respiratory system, cardiovascular system and endocrine system (103). This assessment will allow the person to be framed in order to identify the types of physical activity that can be done safely. It is important to remember how, in addition to these fundamental considerations that are a guarantee of safety for the person, the choice of intervention should also be made respecting the person’s preferences, so as to have his or her greater involvement in the course of treatment and, consequently, greater adherence (173). However, with full respect for the person’s abilities and preferences, group physical activity should be the first choice. In fact, there is evidence that group physical activity leads to benefits not only in the physical domain, but also in the social domain, with a decrease in loneliness’ levels and social isolation (178). Exercise should embrace all the facets, with a part dedicated to muscle strengthening, a resistance training and an aerobic component (177); nevertheless, also programs based on flexibility activities, such as yoga (179) and Tai Chi (102) brings benefits. Individual physical activity should follow the same principles, with the advantage of a wider possibility of customizing the intervention, and with the opportunity to be home-based.

### 6.3 Customized nutritional interventions: personalized nutritional interventions that address individual needs

In order to optimize exercise-related outcomes, a careful assessment of nutritional status is essential (180). In fact, it is well-documented that in older adults both energy and protein intakes are lower than required (181), thereby hindering the benefits of exercise. In this perspective, this type of assessment should be carried out in conjunction with exercise assessment. It is recommended that healthy individuals consume protein at a level of 1.0–1.2 g/kg of body weight (182). In the case of older adults who are malnourished or diagnosed with a chronic disease that may increase the risk of malnutrition, the intake should range from 1.2 to 1.5 g of protein/kg of body weight. Patients suffering from acute illness or severe malnutrition may require a protein intake of up to 2 g/kg of body weight. The amount should be individually adjusted based on nutritional status, physical activity levels, disease condition, and tolerance (94, 95). The recommended daily energy intake for older adults is 30 kcal per kilogram of body weight; however, this value should be individually adjusted based on nutritional status, physical activity level, and health condition (94, 183).

### 6.4 Cognitive and mental health support: cognitive and psychological support tailored to the patient

Physical activity shows benefits also in cognitive functioning (184). Additionally, supplementing physical activity with cognitive stimuli, such as dual-task exercises, showed important improvements in cognitive outcomes (185). However, more specific and focused interventions are needed. Cognitive stimulation, defined as an intervention designed to encourage participation in tasks aimed at improving social and cognitive functioning, is reported to be effective (186). These interventions are also based on activities of daily living (ADLs), in order to enhance the applicability of the trained abilities in the context of everyday life (186). Regarding well-being and anxiety, animal-assisted activities (especially dogs) are very effective, with perceived improvement in quality of life, emotional state and positive emotions (186). Finally, virtual and augmented reality are a promising intervention in counteracting frailty (187, 188).

### 6.5 Monitoring and feedback

As it turns out, interventions to counter frailty are complex. Especially in the early stages, adherence to the individualized intervention program can be challenged by the many difficulties encountered by the person (189). Nevertheless, following the Stages of Change Theory (190), even after passing the initial stages of the new behavior (in this case, the individualized intervention program), the so-called “maintenance” stage is threatened by the absence of tangible and immediate results, unlike the initial stage, where these are present (191). In this perspective, monitoring (included self-monitoring) and feedback are fundamental.

### 6.6 Real-time monitoring and adjustments

During exercise, real-time monitoring is critical. It is first and foremost a safety issue, especially in people with advanced frailty. For this reason, it is important that the activity be carried out in the presence of personnel trained to intervene in case of sudden health problems. However, this is not possible when the exercise is carried out independently by the person at his or her home. While educating people and their caregivers in early recognition of signs and symptoms of exercise-related health issues is still crucial (192), Information and communication technologies (ICTs) are very useful. In particular, physical activity seems to particularly benefits of an ICT component in the intervention, both in the monitoring (180) and in its conduction (193), which allows a more immediate capacity of adjustments when needed. *T*he adoption of ICTs allows the monitoring of progress with a great accuracy, also with the possibility to collect data useful to tailor the intervention to the person. Nevertheless, these data can also represent a “biofeedback” for the person, allowing to adapt in real-time the activities.

### 6.7 Education and support: patient education and empowerment

Education is fundamental when dealing with frailty, and it should be included in the training programs of the involved health personnel (194). There is evidence that lifestyle is strictly related to FS (195), thus often requiring it to be modified. The changes to behavior required are often numerous and complex for the person, and this aspect should be considered in advanced care planning (196). However, although patient education is an integral and fundamental part of advance care planning, there is evidence that it is still underestimated (197). In particular, patient empowerment is very effective, since it can enhance patient understanding, deal with alleged obstacles, and develop patient confidence and communication abilities (198).

### 6.8 Educating patients to better manage FS

As we can see, education still plays a pivotal role in the management of FS. In this context, there is evidence that it is fundamental in building a body of knowledge on the topic, and it contributes in gathering confidence, skills and abilities that lead to the adoption of a healthy lifestyle (199). A recent systematic review showed that the educational areas of intervention mainly concern empowerment, self-care, and health promotion (199). Nevertheless, self-management behaviors, especially those related to physical activity, such as body weight maintaining and increasing frequent physical exercise, are associated with an improvement of the frailty condition (200).

### 6.9 Empowerment through technology: using technology to support patient autonomy

**Figure 3:**
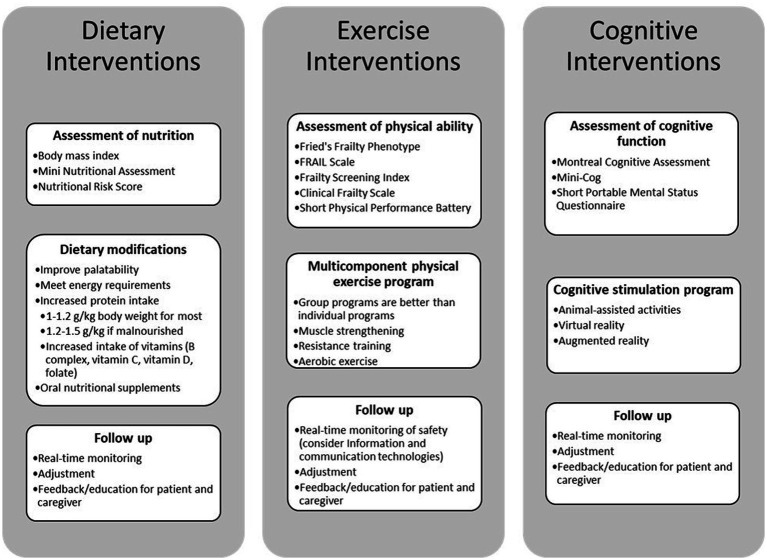
Comprehensive roadmap for integrating frailty interventions into existing care models.

First of all, there is undoubtedly a role for computer-based and online versions of frailty education interventions, given current advancements in digital technology (199). ICTs give not only the ability to monitor the person at home, but also to communicate with them in real time and in a timely manner. In this perspective, it is possible to schedule and perform telemedicine and telenursing visits; to collect relevant information on the frailty status through home automation and sensors (e.g., weight loss, gait speed); and to monitor exercise (including providing live feedback) (201). Figure 3 figure synthesizes the key elements of dietary, exercise, and cognitive strategies, along with their assessment, modifications, and follow-up processes, offering practical guidance for implementation in clinical practice.

## 7 Conclusion and future trends

Integrating personalized interventions into the management of FS in patients with chronic diseases, especially those with cognitive impairments, is essential for improving patient outcomes. However, current strategies face significant limitations. First, a frequent underdiagnosis of cognitive impairments remains, resulting in inadequate care planning. Nevertheless, the lack of standardized protocols, insufficient resources such as specialized personnel and technological tools, and inadequate support for overburdened caregivers further hinder the widespread adoption of individualized care. Additionally, challenges arise from addressing the diverse cultural, social, and socioeconomic backgrounds of patients, limited training among healthcare professionals in recognizing and managing FS and cognitive impairments, ethical and legal considerations related to patient autonomy and consent, financial constraints, and technological barriers among patients and caregivers.

To address these challenges, future research should focus on several key areas. Enhancing the diagnosis of cognitive impairments through more effective screening and assessment tools will enable more accurate care planning. Development of standardized guidelines for more tailored interventions is essential to unify practices across different settings. Improving resource accessibility, including specialized personnel and technological tools, and providing comprehensive support programs for caregivers will help alleviate their burden. Additionally, ensuring that interventions are culturally competent and adaptable will better serve patients from diverse backgrounds. Validating technological innovations, such as AI-based monitoring systems and wearable devices, can offer new opportunities for patient care. Promoting interdisciplinary collaboration among healthcare professionals will foster holistic approaches in patient management. Actively involving patients in their own care through education and shared decision-making improves medication adherence and related clinical outcomes, especially in patients treated with polypharmacotherapy. Integrating community resources can provide additional support, while advocating for healthcare policy reforms can address systemic issues such as funding and resource allocation. Empowering patients through education and self-management strategies can significantly improve their quality of life and promote autonomy in this vulnerable population. Therefore, ongoing research and innovation are pivotal for developing effective, scalable, and adaptable strategies that meet the needs of diverse patient populations.
